# Intravenous Immunoglobulin Therapy for Critically Ill COVID-19 Patients With Different Inflammatory Phenotypes: A Multicenter, Retrospective Study

**DOI:** 10.3389/fimmu.2021.738532

**Published:** 2022-01-27

**Authors:** Yan Chen, Jianfeng Xie, Wenjuan Wu, Shusheng Li, Yu Hu, Ming Hu, Jinxiu Li, Yi Yang, Tingrong Huang, Kun Zheng, Yishan Wang, Hanyujie Kang, Yingzi Huang, Li Jiang, Wei Zhang, Ming Zhong, Ling Sang, Xia Zheng, Chun Pan, Ruiqiang Zheng, Xuyan Li, Zhaohui Tong, Haibo Qiu, Li Weng, Bin Du

**Affiliations:** ^1^ Medical Intensive Care Unit, State Key Laboratory of Complex Severe and Rare Diseases, Peking Union Medical College Hospital, Peking Union Medical College, Chinese Academy of Medical Sciences, Beijing, China; ^2^ Jiangsu Provincial Key Laboratory of Critical Care Medicine, Department of Critical Care Medicine, Zhongda Hospital, School of Medicine, Southeast University, Nanjing, China; ^3^ Department of Critical Care Medicine, Wuhan Jin-Yintan Hospital, Wuhan, China; ^4^ Department of Critical Care Medicine, Tongji Hospital, Tongji Medical College Huazhong University of Science and Technology, Wuhan, China; ^5^ Department of Critical Care Medicine, Union Hospital, Tongji Medical College Huazhong University of Science and Technology, Wuhan, China; ^6^ Department of Infection Disease, Wuhan Pulmonary Hospital, Wuhan, China; ^7^ Department of Critical Care Medicine, Shenzhen Third Hospital, Shenzhen, China; ^8^ Department of Critical Care Medicine, Huangshi Hospital of Chinese Medicine, Huangshi, China; ^9^ Department of Critical Care Medicine, Huangshi Central Hospital, Huangshi, China; ^10^ Department of Respiratory and Critical Care Medicine, Beijing Institute of Respiratory Medicine, Beijing Chao-Yang Hospital, Capital Medical University, Beijing, China; ^11^ Department of Critical Care Medicine, Xuanwu Hospital, Capital Medical University, Beijing, China; ^12^ Emergency Department, The 900th Hospital of Joint Service Corps of Chinese PLA, Fuzhou, China; ^13^ Department of Critical Care Medicine, Zhongshan Hospital, Fudan University, Shanghai, China; ^14^ Department of Critical Care Medicine, Guangzhou Institute of Respiratory Health, The First Affiliated Hospital of Guangzhou Medical University, Guangzhou, China; ^15^ Department of Critical Care Medicine, The First Affiliated Hospital, College of Medicine, Zhejiang University, Hangzhou, China; ^16^ Department of Critical Care Medicine, Northern Jiangsu People’s Hospital, Clinical Medical School, Yangzhou University, Yangzhou, China

**Keywords:** COVID-19, intravenous immunoglobulin therapy (IVIG), hyperinflammation, hypoinflammation, efficiency

## Abstract

**Background:**

The benefits of intravenous immunoglobulin administration are controversial for critically ill COVID-19 patients.

**Methods:**

We analyzed retrospectively the effects of immunoglobulin administration for critically ill COVID-19 patients. The primary outcome was 28-day mortality. Inverse probability of treatment weighting (IPTW) with propensity score was used to account for baseline confounders. Cluster analysis was used to perform phenotype analysis.

**Results:**

Between January 1 and February 29, 2020, 754 patients with complete data from 19 hospitals were enrolled. Death at 28 days occurred for 408 (54.1%) patients. There were 392 (52.0%) patients who received intravenous immunoglobulin, at 11 (interquartile range (IQR) 8, 16) days after illness onset; 30% of these patients received intravenous immunoglobulin prior to intensive care unit (ICU) admission. By unadjusted analysis, no difference was observed for 28-day mortality between the immunoglobulin and non-immunoglobulin groups. Similar results were found by propensity score matching (n = 506) and by IPTW analysis (n = 731). Also, IPTW analysis did not reveal any significant difference between hyperinflammation and hypoinflammation phenotypes.

**Conclusion:**

No significant association was observed for use of intravenous immunoglobulin and decreased mortality of severe COVID-19 patients. Phenotype analysis did not show any survival benefit for patients who received immunoglobulin therapy.

## Introduction

Since March 2020, when the WHO declared the COVID-19 outbreak a global pandemic, the infection caused by severe acute respiratory syndrome coronavirus 2 (SARS-CoV-2) virus has led to 173,674,509 confirmed cases and 3,744,408 deaths to date (1, 2).

Current evidence indicates that severe COVID-19 results from an increased systemic cytokine response and a maladapted host response to the SARS-CoV-2 virus. The aggressive inflammatory response induced by SARS-CoV-2 appears to be associated with lung injury, multiorgan failure, and death (3–5). Because of the specific pathophysiology of COVID-19, immune-based therapies such as intravenous immunoglobulin (IVIG) are under consideration.

However, the effectiveness of IVIG use for COVID-19 patients is controversial. A survival benefit from the administration of IVIG was reported in one single randomized clinical trial (6) and in some observational studies (7–9) but not in others (10–12). The results of a few small trials of IVIG treatment of COVID-19 have not been sufficiently powered to assess differences in mortality (10, 13, 14). Shao et al. conducted a multicenter, retrospective cohort study of IVIG treatment of critically ill COVID-19 patients; the investigators reported significantly reduced 28-day mortality after confounder adjustments (8). However, a weakness of the study was the heterogeneity of the severity of illness among participants, even though the researchers used subgroup analysis of critical and severe patient types. In another retrospective cohort study, Liu et al. did not find any significant difference in mortality of patients who received IVIG compared with non-IVIG patients (12).

COVID-19 populations are heterogeneous (5, 15), which leads to diverse and often ineffective treatment. Accordingly, identifying distinct phenotypes of COVID-19 patients is key to personalized management strategies. Chen et al. used an unsupervised machine learning approach to identify two distinct phenotypes of COVID-19 (16). They found significant survival benefits from corticosteroid treatment of patients with the hyperinflammatory COVID-19 phenotype compared with a hypoinflammatory phenotype. It is unknown whether patients with different COVID-19 phenotypes respond differently to IVIG treatment.

The objective of this study was to assess whether an association existed between IVIG therapy and 28-day mortality of severe COVID-19 patients and to identify COVID-19 patients with hyperinflammatory and hypoinflammatory phenotypes and their responses to IVIG treatment.

## Methods

### Study Design and Participants

We conducted a retrospective cohort study in 19 hospitals in Wuhan (Hubei Province), Huangshi (Hubei Province), Shenzhen (Guangdong Province), and Jiangsu Province. We screened all adult patients with COVID-19 who were admitted to intensive care units (ICUs) of the participating hospitals between January 1 and February 29, 2020. Inclusion criteria were the following: 1) >18 years of age; 2) laboratory-confirmed diagnosis of COVID-19 (17); 3) severe respiratory failure requiring advanced respiratory support (i.e., high flow nasal oxygen, non-invasive mechanical ventilation, and invasive mechanical ventilation), circulatory shock, or multiorgan failure. The Ethics Committee of Jin Yin-tan Hospital approved this study (KY-2020-10.02). Patient-level informed consent was not required because this study was retrospective.

### Data Collection and Outcome

Demographic data, chronic comorbidities, vital signs, and laboratory results obtained within the first 24 h after ICU admission were extracted from electronic medical records. Treatment and outcome data were also recorded. Acute Physiology and Chronic Health Evaluation II (APACHE II) scores were calculated to assess the severity of illness. The main exposure of interest was the administration of IVIG therapy. All data were collected by using a case record form modified from the standardized International Severe Acute Respiratory and Emerging Infection Consort. The primary outcome was 28-day mortality.

### Statistical Analysis

Values were presented as the mean (SD) or median (interquartile range (IQR)) for continuous variables as appropriate and as percent for categorical variables. Comparisons between groups were made using the chi-square test or Fisher’s exact test for categorical variables and Student’s t-test or the Mann–Whitney U test for continuous variables, as appropriate.

### Inverse Probability of Treatment Weighting Using the Propensity Score

Propensity score matching (PSM) was performed to reduce bias by adjusting for the following 15 variables: age; sex; history of hypertension, diabetes, and chronic heart disease; chronic kidney disease, solid malignancy, connective tissue disease, and chronic obstructive lung disease; and vasopressor, invasive mechanical ventilation on ICU admission, APACHE II, disease onset days, and use of glucocorticoids. PSM was implemented with a nearest-neighbor strategy. IVIG and non-IVIG patients were paired according to the propensity scores using exact matching with a caliper size of 0.02 and a paired ratio of 1:1.

During the matching process, a considerable proportion of patients were lost. Thus, inverse probability of treatment weighting (IPTW) using the propensity score analysis was also performed to estimate the causal treatment effects including all eligible patients (entire cohort with complete data on all 15 covariates mentioned above).

The balance of covariates was evaluated by estimating standardized mean differences (SMD) before matching, after matching, and after IPTW adjusted, and a small absolute value less than 0.1 was considered successful balancing between IVIG and non-IVIG patients.

### Association of Intravenous Immunoglobulin Therapy With 28-Day Mortality

Logistic regression and Cox proportional hazards regression were performed to assess an association between IVIG therapy and 28-day mortality. Baseline variables of clinical relevance and significance at the univariable level (p < 0.20) were the following: use of glucocorticoids, APACHE II scores, age, sex, and history of hypertension, diabetes, cardiovascular disease, chronic kidney disease, and chronic obstructive lung disease. The same baseline covariates with IVIG therapy were adjusted in the Cox model. All statistical analyses were performed using R (version 4.0.0, R studio, Boston, MA).

### Cluster Analysis

According to Sinha et al., clinical data such as vital signs and laboratory measurements may enable phenotype identification based on machine learning in acute respiratory distress syndrome (ARDS) (18). As a result, four vital signs (temperature, heart rate, systolic blood pressure, and respiratory rate), eight laboratory measurements (white blood cell, lymphocytes, hematocrit, platelet, sodium, total bilirubin, albumin, and creatine), and two inflammation markers (C-reactive protein and D-dimer) were selected to derive phenotypes. The multiple imputation method was used to account for missing data. The consensus k means clustering models were used to identify COVID-19 phenotypes. Gap statistics was used to determine the optimal number of phenotypes. Logistic regression and Cox analyses before and after IPTW were conducted for each clinical phenotype.

## Results

### Patient Characteristics

In this study, we included 754 critically ill patients, of whom 392 (52.0%) received IVIG therapy. The days from illness onset to ICU admission were longer in the non-IVIG group compared with the IVIG group (13 (IQR 9, 20) vs. 12 (IQR 8, 17), p = 0.009). More patients in the non-IVIG group than in the IVIG group required vasopressin on ICU admission (47% vs. 31%, p = 0.027). Differences in baseline and clinical characteristics are listed in **Table 1**. As seen, SMD of age, history of chronic obstructive pulmonary disease (COPD), vasopressor on ICU admission, illness onset to ICU admission, and glucocorticoid use were more than 0.1. To correct for the potential imbalances, we performed the propensity score-matched method. In the propensity score-matched cohort, 253 patients who received IVIG therapy were matched with 253 non-IVIG therapy patients. During the matching process, 248 individuals were lost. As a result, we performed an additional analysis of the inverse probability of treatment weighting using propensity scores for the entire cohort with complete data on covariates (**Figure S1**).

**Table 1:** Clinical characteristics of 754 patients with COVID-19 included in the study according to IVIG treatment.

Variable	Non-IVIG (n = 362)	IVIG group (n = 392)	p-Value	SMD (%)
Male, n (%)	235 (65)	250 (64)	0.802	2
Age, years	64.5 ± 13.7	63.1 ± 12.5	0.142	11
Comorbidities				
Diabetes, n (%)	70 (19)	75 (19)	1.000	5
Hypertension, n (%)	150 (41)	169 (43)	0.695	3
CHD, n (%)	44 (12)	51 (13)	0.807	3
COPD, n (%)	24 (7)	17 (4)	0.220	10
CKD, n (%)	8 (2)	5 (1)	0.481	7
Malignancy, n (%)	14 (4)	10 (3)	0.412	8
CTD, n (%)	2 (1)	3 (1)	1.000	3
Days from illness onset to hospitalization, median (IQR)	7 (3–11)	6 (3−10)	0.593	4
Days from illness onset to ICU admission, median (IQR)	13 (9–20)	12 (8−17)	0.009	19
APACHE II score^a^	11.3 ± 6.1	10.7 ± 5.6	0.208	9
Organ support on ICU admission				
Invasive ventilation, n (%)	64 (18)	59 (15)	0.380	7
Vasopressor, n (%)	47 (13)	31 (8)	0.027	17
RRT, n (%)	6 (2)	6 (2)	1.000	1
ECMO, n (%)	1 (0)	4 (1)	0.419	9
Laboratory findings on ICU admission				
White blood cell counts, ×10^9^/L	10.3 ± 6.6	10.1 ± 5.9	0.574	4
Lymphocyte count, ×10^9^/L	0.8 ± 1.9	0.8 ± 0.7	0.668	3
Platelet count, ×10^9^/L	178.6 ± 87.9	175.5 ± 77.7	0.619	4
Glucocorticoid treatment, n (%)	175 (48)	301 (77)	<0.001	14
IVIG therapy				
Initiation from onset, median (IQR)	–	11 (8−16)	–	–
Initiation from ICU admission, median (IQR)	–	0 (−2, 1)	–	–
Outcome				
28-day mortality, n (%)	201 (56)	207 (53)	0.499	–

Data presented as n (%) or means ± SD unless otherwise noted. For continuous variables, Mann–Whitney U test was used to calculate the p-value unless otherwise noted. For categorical variables, the chi-square test was used to calculate the p-value unless otherwise noted.

APACHE II, Acute Physiology and Chronic Health Evaluation; CHD, coronary heart disease; CKD, chronic kidney disease; COPD, chronic obstructive pulmonary disease; CTD, connective tissue disease; ECMO, extracorporeal membrane oxygenation; IVIG, intravenous immunoglobulin; RRT, renal replacement therapy; SMD, standardized mean difference.

a The Acute Physiologic Assessment and Chronic Health Evaluation II (APACHE II) score is calculated from 12 measurements during the first 24-h ICU admission. Scores can range from 0 to 71, with higher scores indicating more severe disease.

### Details of Intravenous Immunoglobulin Use

We did not consider the dose and course of IVIG, although most patients were administered with a conventional dose of 0.5 g/kg/day. The patients in the IVIG group received IVIG at 11(IQR 8, 16) days from illness onset (**Table 1**). Prior to ICU admission, 118 of 392 (30.1%) patients received IVIG treatment, while others received IVIG treatment after ICU admission.

### Outcome

**Figure 1:**
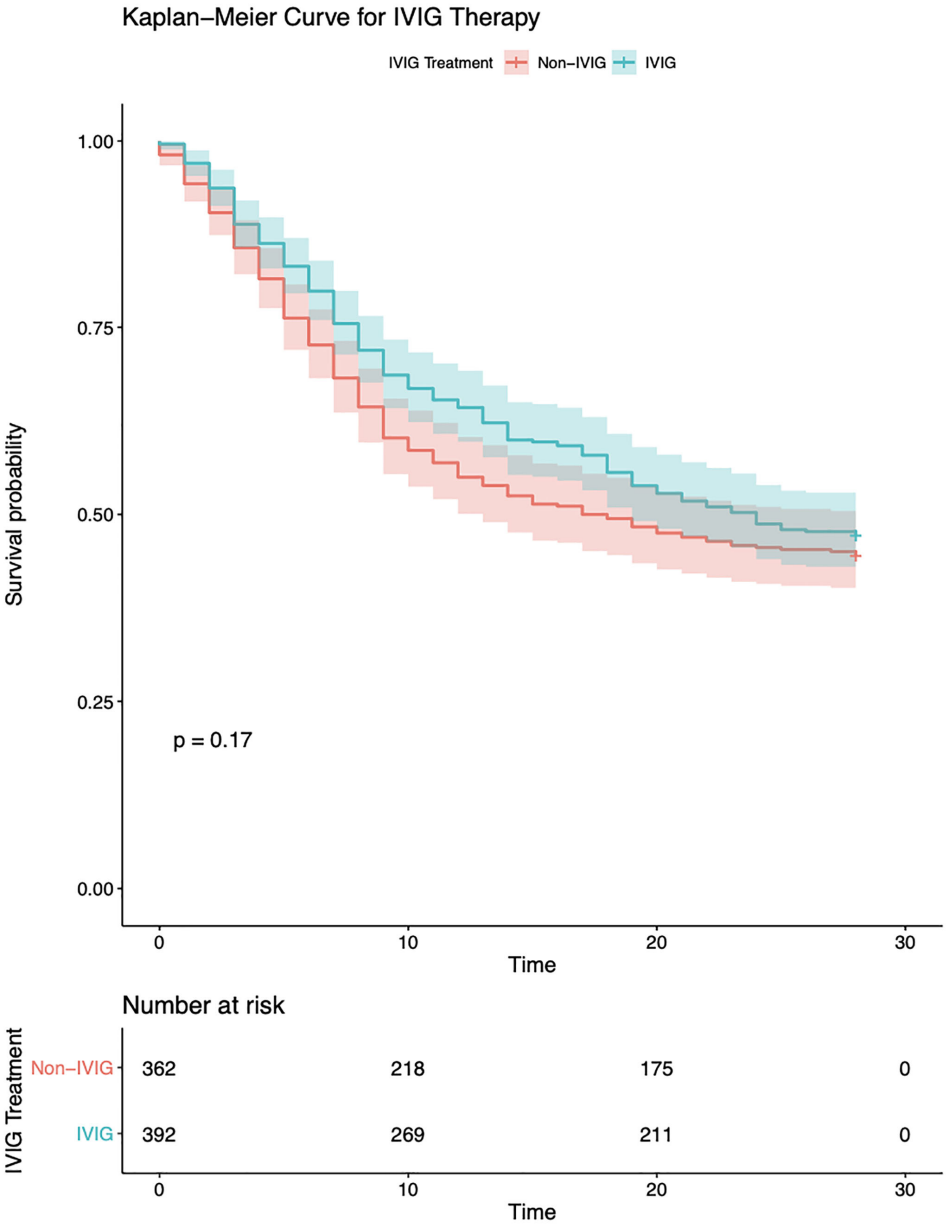
Kaplan–Meier analysis for 28-day survival of the IVIG and non-IVIG groups. IVIG, intravenous immunoglobulin.

The overall cohort 28-day mortality was 54.1%. There were no significant differences between the groups in the number of deaths by 28 days (**Table 1**). Kaplan–Meier curves for estimated survival did not show any significant differences in the outcome (**Figure 1**).

### Propensity Score-Matched Analysis

The PSM resulted in 253 patients who received IVIG matched to 253 patients who did not receive IVIG. More participants received IVIG than those who did not receive it; thus, 139 IVIG patients were unmatched in contrast to 109 non-IVIG patients. **Figure S2** reports the SMD for each of the 15 baseline covariates before and after matching. In propensity score-matched analysis (n = 506), compared with the control group, IVIG therapy was not associated with differences in 28-day mortality, neither in a logistic regression model nor in a Cox model (**Table 2**).

**Table 2:** 28-day mortality of critically ill patients with distinct phenotypes in COVID-19 using various adjustment methodologies.

Cohorts	Logistic regression model^d^		Cox proportional hazards regression model^e^	
	aOR (95% CI)	p-Value	aHR (95% CI)	p-Value
**All patients treated with IVIG vs. patients not treated with IVIG (reference)**				
Original cohort^a^	1.00 (0.70, 1.43)	0.994	0.94 (0.76, 1.15)	0.527
PSM cohort^b^	1.28 (0.86, 1.93)	0.227	1.10 (0.87, 1.38)	0.426
IPTW cohort^c^	0.95 (0.65, 1.37)	0.774	0.94 (0.76, 1.15)	0.534
**Patients with hyperinflammation treated with IVIG vs. patients not treated with IVIG (reference)**				
Original cohort^a^	1.05 (0.57, 1.94)	0.871	0.96 (0.74, 1.25)	0.779
PSM cohort^b^	1.52 (0.77, 3.02)	0.227	1.13 (0.84, 1.51)	0.423
IPTW cohort^c^	1.07 (0.57, 2.01)	0.837	0.98 (0.75, 1.29)	0.911
**Patients with hypoinflammation treated with IVIG vs. patients not treated with IVIG (reference)**				
Original cohort^a^	1.01 (0.63, 1.61)	0.966	0.92 (0.66, 1.28)	0.615
PSM cohort^b^	1.15 (0.67, 1.96)	0.616	0.96 (0.66, 1.40)	0.820
IPTW cohort^c^	0.90 (0.55, 1.46)	0.670	0.87 (0.62, 1.22)	0.427

aHR, adjusted hazard ratio; aOR, adjusted odds ratio; IPTW, inverse probability of treatment weighting; IVIG, intravenous immunoglobulin; PSM, propensity score matching; IPTW, inverse probability of treatment weighting.

a The original cohort was the overall cohort that met inclusion criteria and comprises 392 patients with IVIG therapy and 362 patients with non-IVIG therapy.

b The propensity score-matched cohort comprises 253 patients with IVIG therapy and 253 patients with non-IVIG therapy.

c The entire cohort with complete data on covariates was included in propensity score inverse probability of treatment weighting analysis and comprises 388 patients with IVIG therapy and 343 patients with non-IVIG therapy.

d The logistic regression model was adjusted for the use of glucocorticoids, APACHE II scores, age, sex, and history of hypertension, diabetes, cardiovascular disease, CKD, and COPD.

e The Cox proportional hazards regression model was adjusted for the same abovementioned baseline covariates.

### Inverse Probability of Treatment Weighting Using the Propensity Score

Inverse probability of treatment weighting (IPTW), assessed from patients with complete covariate data included in the propensity analysis, also resulted in between-group balance on baseline characteristics (n = 731). All baseline variables had SMD values of less than 0.1. **Figure S2** reports the SMD for each of the 15 baseline covariates before and after weighting. Both logistic regression model and Cox model did not show a significant difference between IVIG treatment and 28-day mortality, compared with the control non-IVIG group (**Table 2**).

### Cluster Analysis

**Figure 2:**
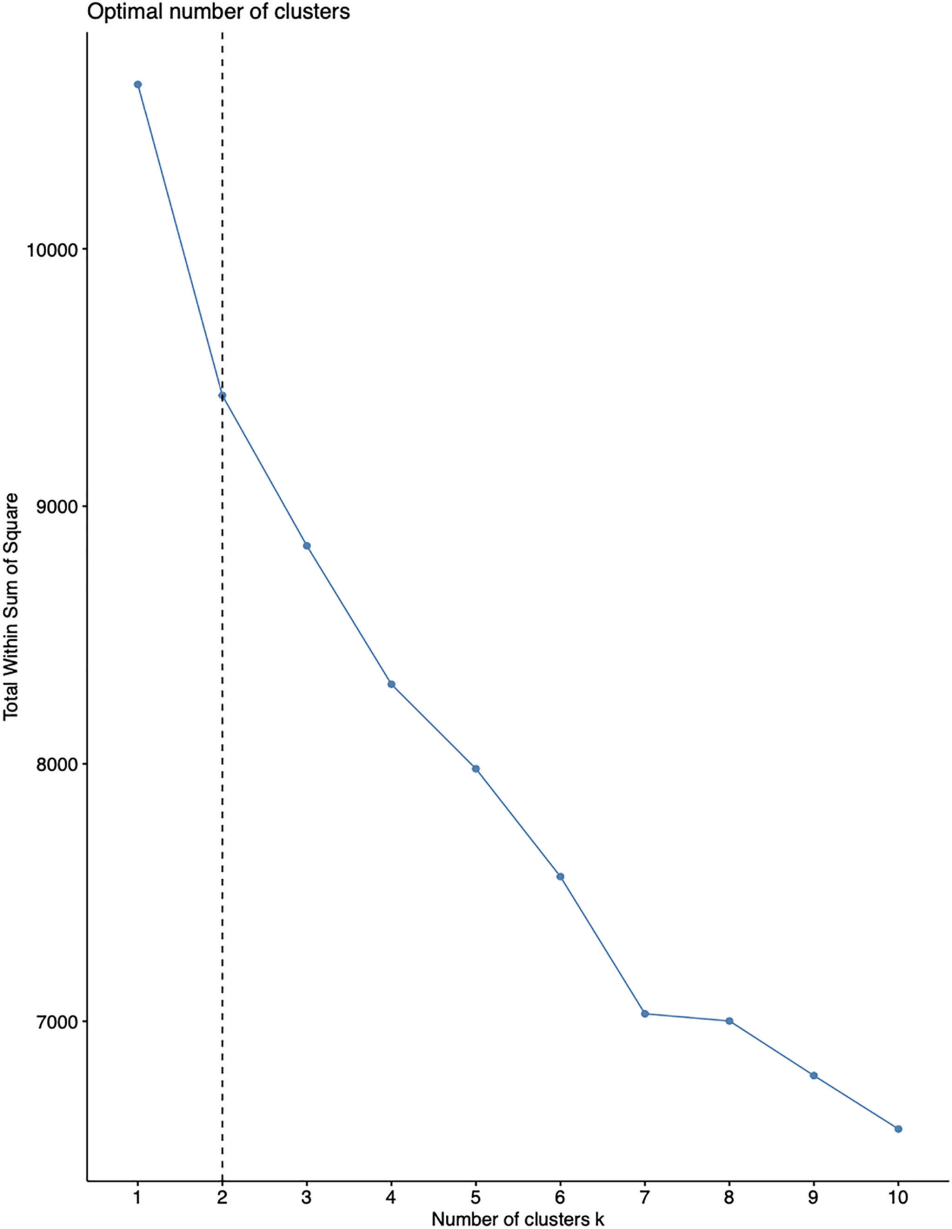
Estimating the optimal number of clusters using gap statistics. The optimal number of clusters was estimated using gap statistics. The optimal number was indicated by the dashed line.

We found by consensus k means clustering models based on clinical data and inflammatory markers that a 2-class model was the optimal fit for the two distinct phenotypes of COVID-19 patients (**Figure 2**). Ultimately, 438 patients were classified as phenotype 1 with less severity and hypoinflammation, and 316 patients were classified as phenotype 2 with more severity and hyperinflammation. We did not find any benefit of IVIG therapy with respect to 28-day mortality for either hyperinflammation or hypoinflammation patients (**Table 2**).

## Discussion

In this retrospective cohort study, we did not find IVIG therapy for COVID-19 associated with a lower risk of 28-day mortality compared with the absence of IVIG. This finding was consistent across analytic approaches and different COVID-19 phenotypes.

As noted by Tang et al., patients with severe COVID-19 are prone to have high concentrations of pro-inflammatory cytokines (19). The dysregulation of immunoreactivity induced by SARS-CoV-2 infection may contribute to disease severity and death (20). IVIG is polyvalent immunoglobulin that includes IgG and trace amounts of IgA, soluble CD4, CD8, HLA molecules, and some cytokines (21). The immunoregulatory effects of IVIG include, but are not limited to, blockade of Fc receptors on immune cells, negative regulation of lymphocyte proliferation, and inflammatory reactions (22). Treatment with IVIG has potential effects of passive immunity and anti-inflammation, which provide a rationale for the use of IVIG in severe infections. IVIG has long been considered a candidate drug for the treatment of severe influenza (23, 24) and coronaviruses such as SARS and Middle East respiratory syndrome (MERS) (25). In a randomized controlled trial of severe influenza A (H1N1), Hung et al. (24) observed reductions in the concentrations of cytokines and viral load and reduced mortality in an IVIG group. According to a retrospective study by Cao et al., administration of high-dose IVIG (2 g/kg) was associated with reduced 28-day mortality (HR 0.24; 95% CI 0.06– 0.99, p < 0.001) in subsets of severe COVID-19 patients within 14 days of onset (9). This finding is different from what we found in this study. We attribute this discrepancy to different sample sizes and different doses and courses of IVIG treatment. Liu et al. (26) suggested that, compared with the regular small dose of IVIG, high-dose IVIG may produce immunomodulatory effects by different mechanisms. Our results are consistent with previous findings in a retrospective, multicenter cohort conducted by Liu et al. (12).

According to Osuchowski et al. (27), SARS-CoV-2 possesses features of both low and high pathogenic coronavirus subspecies, which lead to distinct profiles of clinical and pathophysiological features. As a result, the priority now is to identify subsets of patients who are most likely to benefit from a particular treatment modality. Thus, we endeavored to identify COVID-19 phenotypes based on routine clinical data and inflammation markers. We found two phenotypes distinguished by different levels of inflammation. Individuals with hyperinflammation were prone to worse outcomes than hypoinflammation persons, a result similar to the findings of Manson et al. (28). We then performed phenotype analysis to assess whether the effectiveness of IVIG differed between the two phenotypic groups. Chen et al. reported that glucocorticoid treatment was associated with a reduced hazard ratio for 28-day mortality (HR 0.51; 95% CI 0.34–0.78; p = 0.0018) in a hyperinflammation subgroup (16). However, we did not find a similar superiority of IVIG treatment effectiveness in our hyperinflammation group.

One strength of our study was that it included a multicenter cohort of more than 700 patients with severe COVID-19. The data were collected during the early phase of the COVID-19 outbreak in China. We used robust statistical methodologies such as propensity score matching and inverse probability of treatment weighting to compare 28-day mortality of patients treated or not treated with IVIG. Assessment of IVIG treatment effect was limited in most previous studies because of small sample sizes (<100 participants). The retrospective cohort study conducted by Liu et al. mentioned earlier had a sample size comparable with that of our study (12). However, Liu et al. did not attempt to correlate results with COVID-19 phenotypes. Our study provides clinical outcome data that are likely representative of patients with severe COVID-19 and the subsets of different phenotypes.

Our study also had some limitations. Because of its observational design, unmeasured confounders and residual measured confounders may have influenced the results despite effective propensity score matching and inverse probability of treatment weighting. We did not consider the dose and course of IVIG treatment because the IVIG regimen protocol was not available during the period of COVID-19 disease represented by our cohort; most patients were administered with 0.5 g/kg/day. Also, confounders such as the allocation of medical resources during the emergency may have been associated with mortality but difficult to measure quantitatively in our cohort. Subtle phenotypes may have been missed because of a lack of plasma biomarkers such as interleukin-6 and ferritin. In addition, the safety and tolerance of IVIG therapy were not recorded, although Ferrara et al. have reported minor adverse reactions (29).

## Conclusion

Among severe patients with COVID-19, IVIG therapy was not associated with the lower risks of 28-day mortality, compared with the control group. Phenotype analysis also showed no survival benefits in patients who received IVIG therapy. A randomized clinical trial is needed for the estimation of the benefits of IVIG treatment on COVID-19.

## Data Availability Statement

The raw data supporting the conclusions of this article will be made available by the authors, without undue reservation.

## Ethics Statement

The Ethics Committee of Jin Yin-tan Hospital approved this study (KY-2020-10.02). Patient-level informed consent was not required because this study was retrospective.

## Author Contributions

Among the authors in the list JX, WW, SL, YH, MH, JL, ZT, HQ and BD had the idea for and designed the study; ZT, BD and HQ supervised the study; LW and YC did the statistical analysis. All authors contributed to acquisition, analysis, or interpretation of data. YC, LW, and BD wrote the manuscript. All authors revised the report and approved the final version before submission.

## Funding

This work was supported, in part, by grants from Ministry of Science and Technology of China (2020YFC0843700 and 2020YFC0841300).

## Author Disclaimer

The funding sources had no role in study design, data collection, data analysis, data interpretation, or writing of the manuscript. The corresponding author had full access to all the data in the study and had final responsibility for the decision to submit for publication.

## Conflict of Interest

The authors declare that the research was conducted in the absence of any commercial or financial relationships that could be construed as a potential conflict of interest.

## Publisher’s Note

All claims expressed in this article are solely those of the authors and do not necessarily represent those of their affiliated organizations, or those of the publisher, the editors and the reviewers. Any product that may be evaluated in this article, or claim that may be made by its manufacturer, is not guaranteed or endorsed by the publisher.
