# Supplementary material for: Intravenous Immunoglobulin Therapy for Critically Ill COVID-19 Patients With Different Inflammatory Phenotypes: A Multicenter, Retrospective Study
Source: Front Immunol. 2022 Jan 27;12:738532. doi: 10.3389/fimmu.2021.738532 (PMC8828477; doi:10.3389/fimmu.2021.738532)
Supplement: Supplementary file 1 [file DataSheet_1.docx]

**Supplementary appendix to**

**Intravenous Immunoglobulin Therapy for Critically Ill Patients with two distinct phenotypes in COVID-19: a multicenter, retrospective study**


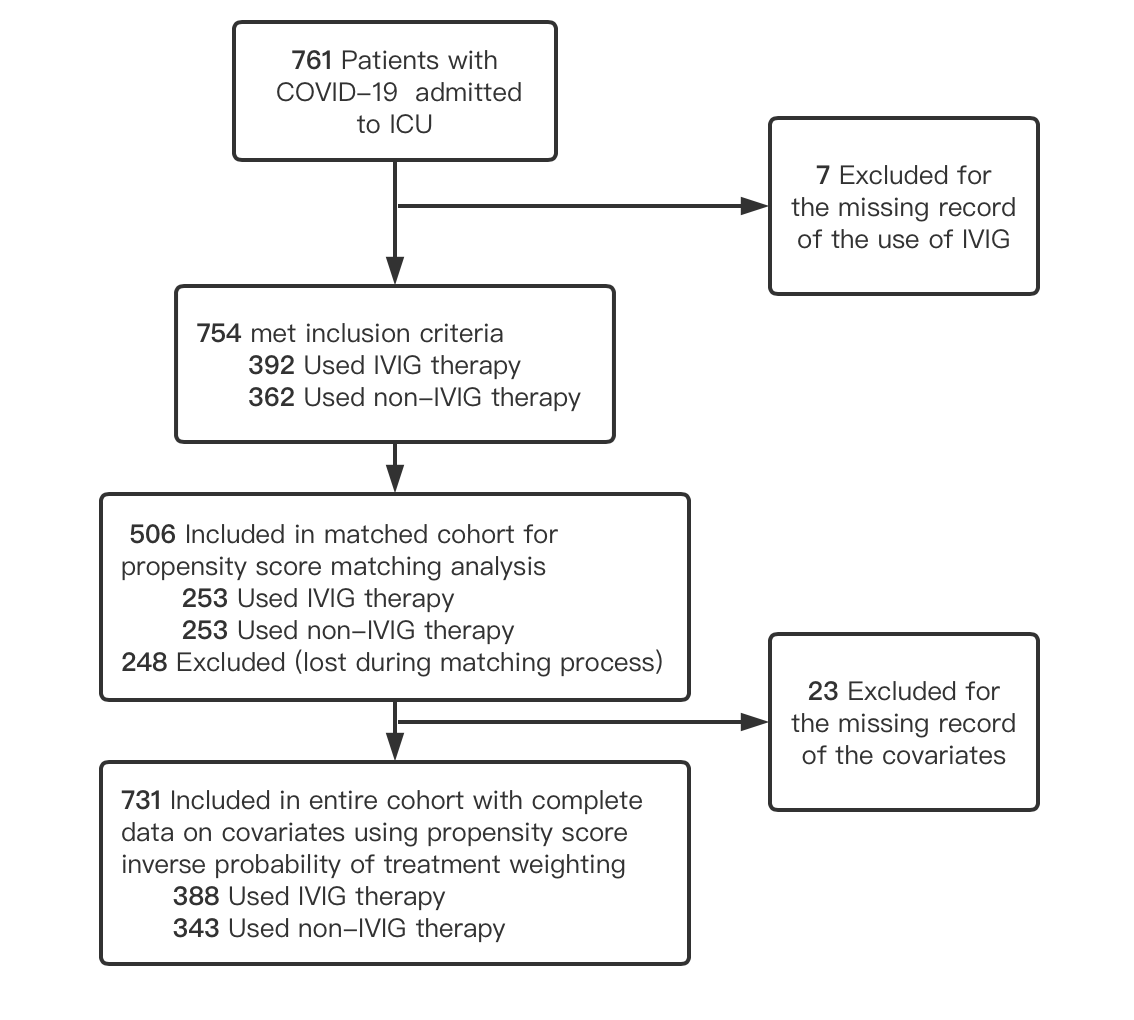


Figure S1. Selection of Study Population

Abbreviations: IVIG, intravenous immunoglobulin


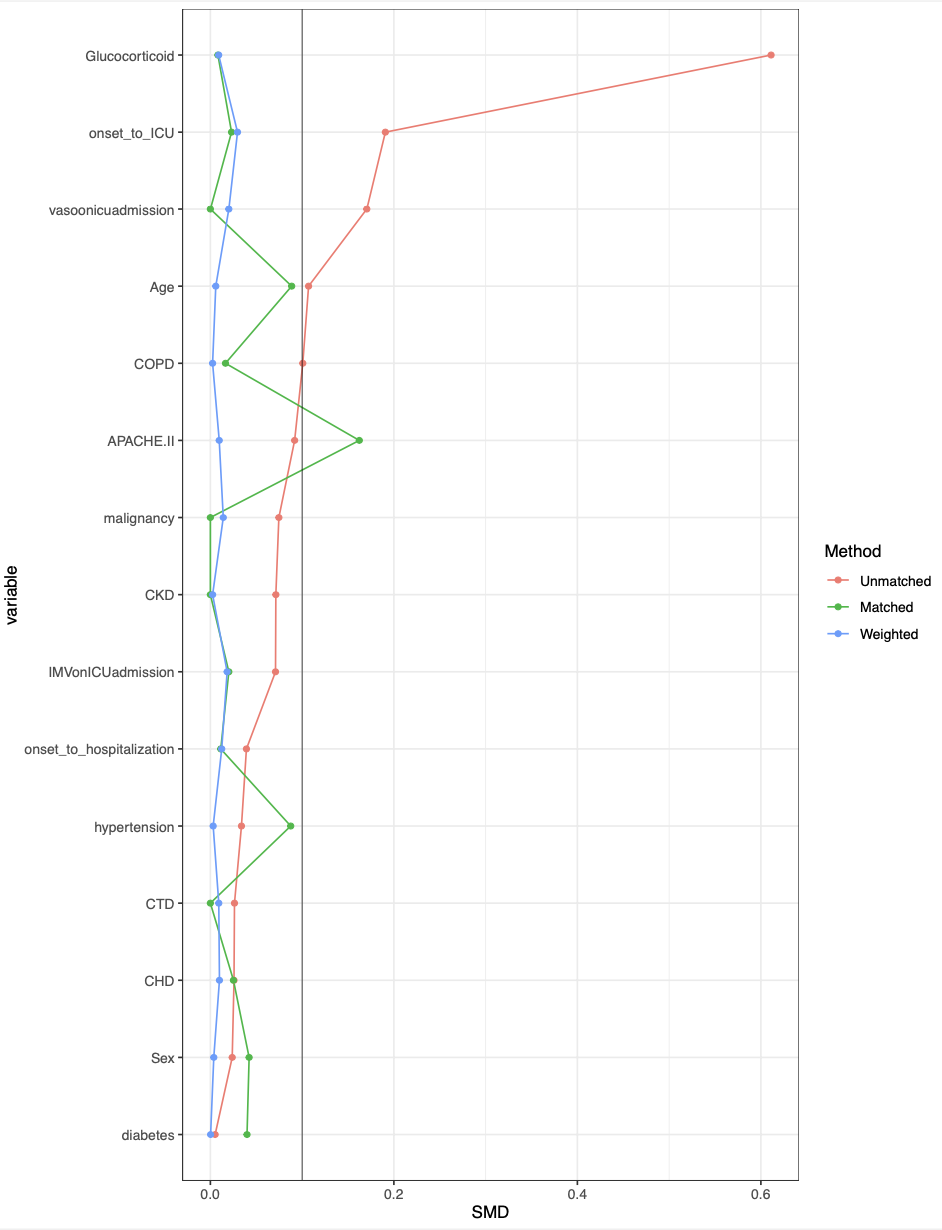


Figure S2. Standardized Mean Difference in Unweighted, Matched and Weighted Cohorts.

Abbreviations: APACHE.II, Acute Physiology and Chronic Health Evaluation; CHD, coronary heart disease; CKD, chronic kidney disease; COPD chronic obstructive pulmonary disease; CTD, connective tissue disease; IMVonICUadmission, invasive mechanical ventilation on ICU admission; onset_to_ICU, illness onset to ICU admission; onset_to_hospitalization, illness onset to hospitalization; SMD, standardized mean differences; vasoonicuadmission, vasopressor on ICU admission.

Method: Unmatched, original cohort; Matched, propensity 1:1 matched cohort; Weighted, inverse probability of treatment weighting cohort.

Small absolute value less than 0.1 was considered successful balancing between the two groups.
